# Integrating Biosimilars Into Oncology Practice: Implications for the Advanced Practitioner

**Published:** 2017-11-01

**Authors:** Christopher J. Campen

**Affiliations:** Greenville Health System Cancer Institute—Pharmacy, Greenville, South Carolina

## Abstract

Biosimilar agents are biologic products that have been shown to be "highly similar" to an already approved reference biologic product. Their integration into clinical practice has the potential to significantly decrease costs for patients, health-care systems, and insurance companies. Through legislation, the US Food and Drug Administration (FDA) approved the Biologics Price Competition and Innovation (BCPI) Act in 2009. In 2010, it was signed into law, allowing for an abbreviated pathway for biosimilar approval. This law implemented a framework for development and regulation of biosimilars for manufacturers and provided guidance for the key submission components necessary to achieve final FDA approval. Many factors will influence how biosimilars are integrated into health-care systems and oncology clinics. As biosimilar utilization in the United States expands beyond supportive care, unique challenges will emerge. Patient and staff education will be at the forefront of the successful application of biosimilar agents in oncology, and advanced practitioners will be in a unique position to lead change. The goal of this article is to describe the chemical and clinical nature of biosimilars, review focus areas of interest for biosimilar development in oncology, discuss implementation strategies for biosimilars, and provide techniques for patient education on biosimilars.

**Integrating Biosimilars Into Oncology Practice: Implications for the Advanced Practitioner**

A continuing education activity for nurse practitioners, PAs, clinical nurse specialists, advanced degree nurses, oncology and hematology nurses, pharmacists, and physicians.

**Release date:** November 16, 2017

**Expiration date:** November 16, 2018

**Expected time to complete activity:** 0.75 hour

**Annenberg Center for Health Sciences at Eisenhower**

39000 Bob Hope Drive, Dinah Shore Bldg.

Rancho Mirage, CA 92270

Voice: 760-773-4500

Fax: 760-773-4513

E-mail: contactce@annenberg.net

**Journal of the Advanced Practitioner in Oncology**

94 N. Woodhull Road

Huntington, NY 11743

Voice: 631-692-0800

Fax: 631-692-0805

E-mail: ckiffer@hbside.com

© 2017, Annenberg Center for Health Sciences at Eisenhower. All rights reserved.

This article is supported, in part, by an educational grant from the following companies: Pfizer, Boehringer Ingelheim. We appreciate that these companies recognize our need for editorial independence in the development of this, and all content that appears in JADPRO. 

**Faculty**

**Christopher J. Campen, PharmD, BCOP**, Greenville Health System Cancer Institute—Pharmacy, Greenville, South Carolina 

## Activity Rationale and Purpose

Over the past two decades, biologics have had a huge impact on the treatment of cancer in multiple tumor types, and in many cases these agents have improved outcomes and survival. In oncology, a growing number of biosimilars have been approved and are in clinical use in Europe, and it is anticipated that many oncology biosimilars will be approved by the US Food and Drug Administration within the next 18 to 24 months. In light of the potential windfall of biosimilars in the pipeline, the need for education must be a priority. Thus, there is a significant need to educate health-care providers about the nature of these products, their manufacturing process, and how they differ from the original parent compounds. 

## Intended Audience

The activity’s target audience will consist of nurse practitioners, PAs, clinical nurse specialists, advanced degree nurses, oncology and hematology nurses, pharmacists, and physicians.

## Learning Objectives

After completing this educational activity, participants should be able to:

- Demonstrate a clear understanding of the chemical nature of biosimilars, and their differences from biologics and generics.
- Identify biosimilars with recent or pending FDA approvals, and recommendations for their clinical use.
- Be aware of biosimilar nomenclature to avoid any confusion between prescribed drug and parent biologic or other biosimilars.
- Understand the importance of pharmacovigilance to identify potential long-term adverse effects.
- Be prepared to clearly explain and discuss the concept of biosimilars with patients and their family members.

## Continuing Education

Statement of Credit—Participants who successfully complete this activity (including the submission of the post-test and evaluation form) will receive a statement of credit.

**Physicians.** This activity has been planned and implemented in accordance with the accreditation requirements and policies of the Accreditation Council for Continuing Medical Education through the joint providership of the Annenberg Center for Health Sciences at Eisenhower, Harborside Medical Education, and the *Journal of the Advanced Practitioner in Oncology*. The Annenberg Center is accredited by the ACCME to provide continuing medical education for physicians.

The Annenberg Center for Health Sciences at Eisenhower designates this enduring activity for a maximum of 0.75 *AMA PRA Category 1 Credits*™. Physicians should claim only the credit commensurate with the extent of their participation in the activity.

**Nurses.** The Annenberg Center for Health Sciences at Eisenhower is accredited as a provider of continuing nursing education by the American Nurses Credentialing Center’s Commission on Accreditation. 

A maximum of 0.75 contact hour may be earned for successful completion of this activity. 

Provider approved by the California Board of Registered Nursing, Provider No. 13664, for 0.75 contact hour.

**Pharmacists.** The knowledge-based accredited educational activity is intended for pharmacists involved in the care of cancer patients. This educational activity is provided by the Annenberg Center for Health Sciences at Eisenhower. 

The Annenberg Center for Health Sciences at Eisenhower is accredited by the Accreditation Council for Pharmacy Education as a provider of continuing pharmacy education. This program is assigned ACPE Universal Program #0797-9999-17-099-H01-P. This program is designated for up to 0.75 contact hour (0.075 CEU) of continuing pharmacy education credit.

## Financial Disclosures

All individuals in positions to control the content of this program (eg, planners, faculty, content reviewers) are expected to disclose all financial relationships with commercial interests that may have a direct bearing on the subject matter of this continuing education activity. Annenberg Center for Health Sciences at Eisenhower has identified and resolved all conflicts of interest in accordance with the ACHS policies and procedures. Participants have the responsibility to assess the impact (if any) of the disclosed information on the educational value of the activity. 

## Disclosures

**Faculty**

**Christopher J. Campen, PharmD, BCOP**, has received consulting fees/honoraria from Astellas. 

**ANNENBERG CENTER FOR HEALTH SCIENCES AT EISENHOWER**

**John Bayliss**, VP, Business Development, spouse is an employee of Amgen, Inc; Charles Willis, Director, Continuing Education, consults for Pfizer Inc.; all other staff at the Annenberg Center for Health Sciences at Eisenhower have no relevant commercial relationships to disclose.

**Lead Nurse Planner**

**Dorothy Caputo, MA, BSN, RN**, has nothing to disclose.

**Planners**

**Jeannine Coronna** has nothing to disclose.

**Claudine Kiffer** has nothing to disclose.

**Sarah McGullam** has nothing to disclose. 

**Lynn Rubin** has nothing to disclose. 

**Lisa Ryan** has nothing to disclose. 

**Pamela Hallquist Viale, RN, MS, CNS, ANP**, has nothing to disclose.

**Annie Yueh** has nothing to disclose.

**Content Reviewers**

**Kelley D. Mayden, MSN, FNP, AOCNP®, IAC**, has received consulting fees/honoraria and has served on the speakers bureau for Puma Biotechnology, and has also served on the speakers bureaus for Apobiologix and Takeda.

## Disclaimer

This activity has been designed to provide continuing education that is focused on specific objectives. In selecting educational activities, clinicians should pay special attention to the relevance of those objectives and the application to their particular needs. The intent of all Annenberg Center for Health Sciences at Eisenhower educational opportunities is to provide learning that will improve patient care. Clinicians are encouraged to reflect on this activity and its applicability to their own patient population.

The opinions expressed in this activity are those of the faculty and reviewers and do not represent an endorsement by Annenberg Center for Health Sciences at Eisenhower of any specific therapeutics or approaches to diagnosis or patient management.

## Product Disclosure

This educational activity may contain discussion of published as well as investigational uses of agents that are not approved by the US Food and Drug Administration. For additional information about approved uses, including approved indications, contraindications, and warnings, please refer to the prescribing information for each product.

## How to Earn Credit

To access the learning assessment and evaluation form online, visit http://surveys.edmeasures.com/s3/Biosimilars-Print-Post-Test

**Statement of Credit:** Participants who successfully complete this activity (including scoring of a minimum of 70% on the learning assessment) and complete and submit the evaluation form will be able to download a statement of credit.

## Integrating Biosimilars Into Oncology Practice: Implications for the Advanced Practitioner

Biologic agents play an increasingly important role in the medical care of patients. Between 2010 and 2015, biologics accounted for 22% of drugs approved by the US Food and Drug Administration (FDA). This comes at a cost, however: Although less than 1% of all prescriptions dispensed in the United States are biologics, 28% of prescription drug spending is associated with biologic agents (Sarpatwari, Avorn, & Kesselheim, 2015). By 2019, it is estimated that the global market for biologics will reach $66.4 billion (Camacho & Pai, 2015).

As such, there is a pressing need to reduce medication costs to manage health-care resources responsibly. One way of accomplishing this is through the development of pathways for generic drug approval. In 1984, Congress passed the Drug Price Competition and Patent Term Restoration Act (Hatch-Waxman Act), which applied a process for generic medication approval to chemical agents. It is estimated that in 2015, 89% of prescriptions were filled for generic medications, accounting for a cost savings of $227 billion (Generic Pharmaceutical Association, 2016).

The Hatch-Waxman Act applied to relatively simple chemical structures where manufacturing processes could make exact duplicates of reference medicines. The Act did not apply to biologic agents due to the complexity involved in the development and manufacturing of biologic agents; this resulted in a gap in the development of products that were near identical—or biosimilar—to a licensed biologic product. The European Medicine Agency (EMA) was the first regulatory agency to approve biosimilar medications, with the introduction of biosimilars for epoetin alfa in 2007 and filgrastim in 2008. The United States, however, has lagged years behind. The Biologics Price Competition and Innovation (BCPI) Act was introduced through legislation in 2009. In 2010, it was signed into law as part of the Patient Protection and Affordable Care Act, allowing for an abbreviated pathway for biosimilar approval (Christl, 2015).

## UNDERSTANDING BIOLOGICS, BIOSIMILARS, AND GENERIC DRUGS

Biologic therapies are highly complex medicines created from living cells, in contrast to small-molecule drugs, which have relatively simple chemical structures and a more straightforward manufacturing process. Table 1 compares biosimilar and generic drugs with regard to their molecular structures and pathways to FDA approval. Examples of biologic agents include vaccines, gene therapies, and monoclonal antibodies. Biologic structures can consist of proteins, sugars, nucleic acids, or a combination of the three.

**Table 1:**
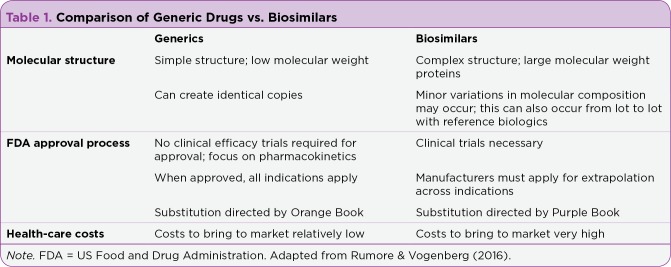
Comparison of Generic Drugs vs. Biosimilars

Due to the complexity involved in creating biologics, heterogeneity in the final product may occur. Heterogeneity refers to minor variations in the biologic agent that can occur both naturally or through the production process. These differences can contribute to batch-to-batch variations that are expected in the development of complex biologics. An increased level of inspection and scrutiny of manufacturing processes is therefore necessary to ensure patient safety and clinician trust of biosimilar development (Schellekens, Smolen, Dicato, & Rifkin, 2016). 

The term "biosimilar" was first established by the European Union in 2006 to describe biologics, which are similar copies of reference agents. Table 2 defines common terms that are used in the context of biosimilar agents.

**Table 2:**
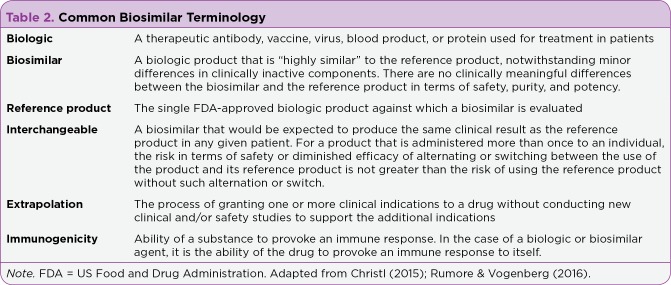
Common Biosimilar Terminology

In the United States, the FDA defines biosimilarity using two core criteria. First, the biologic product must be "highly similar" to the corresponding reference biologic, in that there may be minor differences in the production process or clinically inactive components (Christl, 2015). Second, there should be "no clinically meaningful differences between the biological product and the reference product in terms of the safety, purity, and potency of the product."

The FDA reviews the "totality of evidence" to determine whether a biologic agent is biosimilar to the reference agent to which it is being compared (Christl, Woodcock, & Kozlowski, 2017). The minor differences that can occur in the development and production of biosimilars point to the necessity for a complex framework for review and approval by the FDA, which incorporates analytic, pharmacologic, and clinical data. This framework for FDA review of biosimilars was created to ensure that patients and providers are confident in both the safety and efficacy of a biosimilar agent similar to a reference biologic agent.

There are unique requirements for the FDA approval of a biosimilar agent vs. the original reference biologic agent. The biosimilar must have the same dosage form, concentration, and route of administration as the reference biologic agent. The manufacturing facility must pass rigorous inspections and review by the FDA. A strong emphasis is placed on analytic studies, which are the foundation for potential approval of a biosimilar agent. The goal of these analytic studies is to show that the agent is "highly similar" to the reference biologic agent. Depending upon the analysis of data by the FDA, the level of nonclinical and clinical studies for submission will be decided.

Initial animal and human studies must be performed to assess toxicities and pharmacokinetics/pharmacodynamics. Large clinical studies can add to the level of data and reduce uncertainty that may be present within the analytic data (Christl et al., 2017). The FDA does not require clinical studies for all indications for which the manufacturer may be seeking FDA approval. In oncology this is important, as many reference biologics have lost or are soon to lose patent approval, including rituximab, trastuzumab, bevacizumab, and cetuximab (Rugo, Linton, Cervi, Rosenberg, & Jacobs, 2016). Clinical studies can also help define any risk of immunogenicity, which is the most significant safety concern within biosimilar development and clinical use (Liu, Zou, Sadhu, Shen, & Nock, 2015). 

## COST IMPLICATIONS OF BIOSIMILAR APPROVAL

Over the past 10 years, patent expiration of biologic agents in oncology has led to significant interest in the development of biosimilar agents. This includes monoclonal antibodies for therapeutic indications in oncology along with biologic products for supportive care indications. In 2015, the first biosimilar, filgrastim-sndz, was approved in the United States. Filgrastim-sndz is a biosimilar for the reference product filgrastim, a short-acting granulocyte-colony stimulating factor (G-CSF). Within 4 months of FDA approval, filgrastim-sndz attained 24% of the short-acting G-CSF market after pricing an initial 15% average wholesale price (AWP) discount compared with filgrastim (Blank, 2017).

The first FDA-approved biosimilar for cancer treatment is bevacizumab-awwb (US Food and Drug Administration, 2017a). Pricing and a product launch time for bevacizumab-awwb has not been set at this time due to a patent expiration of 2019 for bevacizumab. 

Five other biosimilars have been approved by the FDA for non-oncologic indications. They include two biosimilars for infliximab, two biosimilars for adalimumab, and one for etanercept (Center for Drug Evaluation and Research, 2017); these biosimilars are primarily used for autoimmune disorders. In the case of infliximab, there are now three therapeutic options for providers (one reference agent and two biosimilars), which has resulted in a significant improvement in pricing for patients. One biosimilar, infliximab-abda (Renflexis), was initially priced at a 35% AWP discount to the reference product infliximab, demonstrating the impact of free-market competition in decreasing costs for patients, health systems, and society (George, 2017). 

## BIOSIMILARS IN ONCOLOGY

There has been significant interest in the development of biosimilars in oncology due to recent or pending patent expirations of several core biologic agents. They include epoetin alfa, pegfilgrastim, rituximab, trastuzumab, bevacizumab, and cetuximab. Table 3 describes recent updates in this area of biosimilar development.

**Table 3:**
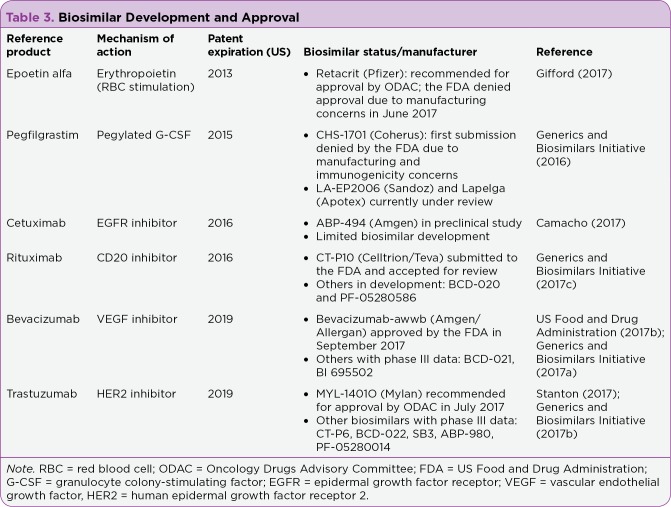
Biosimilar Development and Approval

The field of biosimilar development has quickly become crowded. For example, three biosimilars for trastuzumab have been submitted to the FDA, with one recommended for approval by the FDA Oncology Drugs Advisory Committee (ODAC; Stanton, 2017). There are currently at least six biosimilars for trastuzumab with phase III data reported (Generics and Biosimilars Initiative, 2017b). Trastuzumab does not lose patent protection until 2019 in the United States, and it is expected that even more biosimilars could have additional data or be reviewed for approval by the FDA in the interim (Rugo et al., 2016). As described earlier in this article, the data submitted to the FDA for approval are based on the "totality of evidence" comprising analytic, nonclinical and clinical pharmacokinetics and pharmacodynamics, and clinical study results (Christl et al., 2017). In the case of oncologic indications, where the bar is set high for approval, clinical evidence often involves at least one phase III study (Rugo et al., 2016). Of note, the FDA will allow for extrapolation across indications if the clinical data submitted indicate biosimilarity (Christl et al., 2017). 

Phase III results for a trastuzumab biosimilar (MYL-1401O) vs. trastuzumab were recently published (Rugo et al., 2017). In this multicenter, double-blind, randomized study, 500 patients were randomized 1:1 to receive MYL-1401O or trastuzumab plus a taxane for HER2-positive metastatic breast cancer. The overall response rate (ORR) was 69.6% for MYL-1401O vs. 64% for trastuzumab, which was not statistically significant. Other measures, including progression-free survival, time to tumor progression, and overall survival, were not statistically significant at 48 weeks of follow-up. No difference was seen in adverse effects, including cardiac changes. Other biosimilar agents to trastuzumab have reported phase III data and are currently in the process of FDA review (Generics and Biosimilars Initiative, 2017b).

Several biosimilars for bevacizumab are also in the late stages of development and submission to the FDA. Recently, bevacizumab-awwb was approved by the FDA based on preclinical and clinical data (US Food and Drug Administration, 2017a). In a phase III study, bevacizumab-awwb was compared with bevacizumab at the equal 15-mg/kg dose in patients with nonsquamous non–small cell lung cancer receiving carboplatin and paclitaxel administered every 3 weeks (Thatcher et al., 2016). A total of 328 patients received bevacizumab-awwb and 314 patients received bevacizumab. The ORR was 39% vs. 41.3% for bevacizumab-awwb and bevacizumab, respectively. The difference was not statistically significant. No difference in adverse effects was reported. Binding antibodies developed in 1.4% of bevacizumab-awwb patients and 2.5% of bevacizumab patients. 

Rituximab is now off patent, meaning a biosimilar agent could be approved for use and introduced to the market at this time. The biosimilar CT-P10 has now been accepted for review by the FDA (Generics and Biosimilars Initiative, 2017c). A phase III study compared CT-P10 and rituximab when combined with cyclophosphamide, vincristine, and prednisone (CVP) in newly diagnosed advanced-stage follicular lymphoma (Kim et al., 2017). A total of 140 patients were randomized in a 1:1 ratio. The overall response rate was 97% for patients receiving CVP plus CT-P10 vs. 92.6% for patients receiving CVP plus rituximab. No statistically significant differences in adverse events were observed. Binding antibodies occurred in 4.3% of patients receiving CT-P10 vs. 2.9% in patients receiving rituximab. 

## RISK OF IMMUNOGENICITY

Immunogenicity is an immune-related response or immune reaction of the host against a therapeutic protein. This is a common occurrence after the introduction of therapeutic proteins in the body; however, clinical impact is rare. The development of immunogenicity can be both cellular and humoral and may be complex. Immunogenicity can be measured through the development of antibodies targeting the therapeutic protein (anti-drug antibodies). This process can occur in the context of biologic or biosimilar drug use due to the complex process in drug development (Pedras-Vasconcelos, 2014). The greatest concern in the context of biosimilar drug development is the risk of an immune reaction if the patient is switched back and forth between a reference agent and the biosimilar product due to a higher theoretical risk of immunogenicity (Markus et al., 2017).

Clinical adverse effects from immunogenicity may arise either due to an alternation in pharmacologic activity or from an immune-mediated toxicity during long-term treatment (Clarke, 2010). This can mechanistically occur through binding antibodies to therapeutic proteins or neutralizing antibodies that impede the activity of the therapeutic protein (Pedras-Vasconcelos, 2014). Altogether, the clinical impact may be a change in pharmacokinetic activity, reduced efficacy, infusion-related reactions, and cross-reactivity to the endogenous counterpart of the therapeutic protein. A loss or change in efficacy is not necessarily linked to an increase in adverse events. Despite these concerns, clinical experience in areas of the world where biosimilars have been integrated for many years has shown safety in long-term follow-up. In Europe, biosimilars have been used for approximately 400 million patient days, with no serious safety signals noted thus far (Schellekens et al., 2016). 

Clinical examples of immunogenicity occurring with therapeutic proteins have been reported in the literature. One often-discussed example was a case of cross-reactivity to the endogenous counterpart of the therapeutic protein epoetin alfa (Eprex). In this case, a change in manufacturing processes and not the production of the therapeutic protein itself resulted in immunogenicity (Pedras-Vasconcelos, 2014). In another case, the incidence of pure red cell aplasia (decrease in red blood cells) was found to have significantly increased outside the United States in patients receiving epoetin alfa between 1998 and 2001 (Bennett et al., 2004). Pure red cell aplasia can be severe, and transfusional support and immunosuppressant treatment were required in many patients.

A root-cause analysis determined that clinically neutralizing antibodies against natural erythropoietin were found in patients who primarily received the Eprex formulation of epoetin alfa. A vehicle formulation change that occurred in 1998 (from the use of albumin to polysorbate 80) was discovered to be the primary cause of the development of this pure red cell aplasia, which reportedly affected 175 patients worldwide. Of note, the onset was not immediate, with a median duration of treatment of 9.1 months in patients receiving Eprex. 

The FDA has placed a strong emphasis on reviewing the risk of immunogenicity and will not allow biosimilars to be approved if there are unanswered questions when reviewing immunogenicity data. Although analytic methodology is a core focus for development and review of biosimilars, risks of immunogenicity cannot be fully determined using analytic methods (Christl et al., 2017). Clinical studies must be performed to assess this risk; in some cases, this can be integrated into other studies, but often immunogenicity risk must be the focus of the study.

When reviewing biosimilars for approval, the FDA also does not focus solely on the data; the agency will also review manufacturing facilities and processes to determine whether the entire application is with merit. A recent example of this in the United States involved another biosimilar to epoetin alfa (Gifford, 2017). In this case, ODAC recommended to approve the biosimilar agent manufactured by Pfizer based on the analytic and clinical data submitted. The FDA, however, denied the approval of the biosimilar, citing concerns with the manufacturing facility and process. The FDA did not request additional clinical data. This underscores the importance of a safe manufacturing process and stresses the high bar for safety being set by the FDA. 

## INTERCHANGEABILITY

All currently approved biosimilars can be prescribed in the same way any medication would be written. The details of the prescription must include the exact name, and no substitutions are permitted. For example, if a prescriber wrote a prescription for filgrastim, at this time it would be dispensed as the corresponding biologic agent, filgrastim. If the prescriber wanted to have a biosimilar dispensed, the written name would be filgrastim-sndz. At this time, filgrastim-sndz is not considered interchangeable with the reference product filgrastim. 

There are two additional requirements in the United States for a biosimilar to be designated by the FDA as interchangeable with a reference product. Defined by the FDA, the biosimilar would be "expected to produce the same clinical result as the reference product in any given patient; and for a product that is administered more than once to an individual, the risk in terms of safety or diminished efficacy of alternating or switching between use of the product and its reference product is not greater than the risk of using the reference product without such alternation or switch" (Christl, 2015). To provide guidance on this issue, the FDA has created the Purple Book, which gives clarity to whether a medication is a biosimilar or additionally interchangeable with a reference biologic (US Food and Drug Administration, 2017b). 

Once a biosimilar is approved as interchangeable, pharmacist substitution is allowed without the intervention of a health-care provider (Christl, 2015). Whether or not manufacturers will pay for additional studies to achieve a designation of interchangeable remains to be seen. At this time, no biosimilars have received FDA approval as being interchangeable; despite this, laws regarding interchangeability have been implemented at the state level, which has created confusion (Rumore & Vogenberg, 2016; US Food and Drug Administration, 2017b). In a situation where the biologic is dispensed frequently from a retail pharmacy, having a designation as interchangeable may be beneficial.

For most biologic agents in oncology, dispensing occurs within a health-system (clinic or hospital) pharmacy; in such cases, a pharmacy and therapeutics medical committee would be expected to review individual biosimilar agents for clinical and financial benefits to determine whether the biosimilar should be added to the formulary (Rumore & Vogenberg, 2016). Systematic changes can occur through mechanisms such as this, and clarity for prescribing practices will require the use of the electronic medical records for guidance. In this situation, a pharmacist would not be substituting a biosimilar agent; rather, the electronic medical system would be guiding the provider to the pharmacy and therapeutics committee–approved biosimilar agent. 

## BARRIERS TO IMPLEMENTATION

There will be numerous barriers to implementation of biosimilar agents. One primary obstacle concerns patient and physician perception of biosimilars. A study performed by the National Comprehensive Cancer Network (NCCN) in 2011 demonstrated that practitioners had moderate-to-high overall interest in prescribing biosimilars; however, approximately 25% of respondents indicated they would need additional information before deciding their future interest in prescribing biosimilars (Zelenetz et al., 2011). Long-term follow-up of data from the integration of biosimilars into practice in Europe and the strict standards set by the FDA will help improve perceptions over time, but education will be vital to reduce the risk of misconceptions regarding efficacy and safety. Lawsuits between manufacturers have delayed the integration of biosimilars in the United States (Rumore & Vogenberg, 2016).

Another barrier is the magnitude of cost benefit for patients and oncology practices. If the decrease in price is minor, the cost of implementation, electronic medical record changes, and education can be a deterrent. To prevent switching between the reference biologic and biosimilar, processes will be required to track use. Unless a complete switch is utilized over time, the pharmacy will need to stock both the reference biologic and biosimilar, which will increase the costs of implementation. If multiple biosimilar agents are approved for a reference agent, record-keeping will be necessary to diminish the risk of switching biosimilar agents in the same patient over time. 

## BIOSIMILAR EDUCATION AND PHARMACOVIGILANCE

One of the core challenges to successfully implementing biosimilars is increasing provider and patient confidence in the efficacy and safety of these agents. Advanced practitioners (APs) can perform a vital role in the management of biosimilars. This starts with AP involvement in clinical studies assessing biosimilar efficacy and safety and continues with education and treatment monitoring. Investigational oncology pharmacists can be involved in biosimilar study design and management, and nurse practitioners and physician assistants in oncology can manage and monitor patients on biosimilar studies and provide patient education.

Education will be necessary for informed decision-making regarding the perceived efficacy and safety of biosimilar agents to improve clinic and hospital adoption and patient confidence. Examples of such education include patient, physician, AP, nursing, and nonclinical staff education. Patient teaching of new treatment regimens or a change in the treatment regimen is often performed by APs. Advanced practitioners will also be involved in the training of medical staff, including physicians and nurses. And, as APs often work for pharmacy and therapeutics committees and related formulary management within hospital systems, they may also be involved in biosimilar clinical and cost review, decision-making analysis, and biosimilar implementation. 

Biosimilars in oncology are currently utilized for supportive care indications. There will be a significantly increased demand for patient and staff education in the future with the pending approvals and/or upcoming patent expirations for oncology biosimilars with therapeutic use, where the bar for efficacy and safety is set extremely high. To successfully implement biosimilar agents within oncology systems, formalized education processes will be necessary. Staff and patients will likely require reassurance that the rigorous process set by the FDA for approval will ensure "no clinically meaningful differences in safety, purity, and potency" between the biosimilar and reference biologic drug (Christl et al., 2017). Additional requirements for clinical data (often at least one phase III comparative study) should also be discussed with staff and patients. In the case of specific biosimilar agents, education including a review of the studies performed will help reassure staff of the clinical rigors required by the FDA. 

Risks of immunogenicity have been a concern, and education needs to include the key steps the FDA and manufacturers take to eliminate this risk. First, preclinical and clinical data are compiled to assess for immunogenicity. Second, postmarketing pharmacovigilance programs have been created to assess any long-term signals that could develop. Advanced practitioners will play a key role in the identification of any unexpected reactions. Staff and patients need to be reassured that biosimilars have been used for approximately 400 million patient days over a 10-year period, and no serious safety signals have been noted (Schellekens et al., 2016). 

## CONCLUSION

The introduction of biosimilar agents in oncology will provide unique opportunities and challenges for APs. Biosimilar agents have the potential to increase access and significantly decrease costs to patients and medical systems. The educational requirements, pharmacy management, and pharmacovigilance efforts could require additional clinic or hospital resources for successful utilization of biosimilars in oncology. For AP education of patients, a clear understanding of the process for biosimilar regulatory approval, including the strict efficacy and safety requirements set by the FDA, will be essential.

**Disclosure**

Dr. Campen has received consulting fees/honoraria from Astellas Pharma.

**How to Earn Credit**

To access the learning assessment and evaluation form online, visit http://surveys.edmeasures.com/s3/Biosimilars-Print-Post-Test

**Tips from the APSHO Experts**

If you are interested in 2-minute videos on this topic, visit http://advancedpractitioner.com and look for this feature in our video offerings!
